# Programs Using Stimulation-Regulating Technologies to Promote Physical Activity in People With Intellectual and Multiple Disabilities: Scoping Review

**DOI:** 10.2196/35217

**Published:** 2022-04-07

**Authors:** Giulio E Lancioni, Nirbhay N Singh, Mark O’Reilly, Jeff Sigafoos, Gloria Alberti, Lorenzo Desideri

**Affiliations:** 1 Department of Neuroscience and Sense Organs University of Bari Bari Italy; 2 Department of Psychiatry and Health Behavior Augusta University Augusta, GA United States; 3 Department of Special Education University of Texas at Austin Austin, TX United States; 4 School of Education Victoria University of Wellington Wellington New Zealand; 5 Lega F D'Oro Research Center Osimo Italy; 6 Department of Psychology University of Bologna Bologna Italy

**Keywords:** technology, intellectual disabilities, sensory impairments, multiple disabilities, physical activity, video games, exergames, response-contingent stimulation, mobile phone

## Abstract

**Background:**

People with intellectual and multiple disabilities tend to engage in very low levels of physical activity.

**Objective:**

This review paper aims to provide a comprehensive picture of intervention programs using stimulation-regulating technologies to promote forms of physical activity in people with intellectual and multiple disabilities.

**Methods:**

Following the PRISMA-ScR (Preferred Reporting Items for Systematic Reviews and Meta-Analyses extension for Scoping Reviews) checklist, a scoping review was conducted to identify and provide a synthesis of eligible studies published in English between 2010 and 2021. Studies were identified by searching PubMed, Web of Science, PsycINFO, ERIC, and CINAHL as well as by using Google Scholar and manual searches. Studies were included if they involved individuals with intellectual or multiple disabilities, used stimulation-regulating technology systems to help participants engage in physical activity, and reported data on the impact of the intervention.

**Results:**

A total of 42 studies met the inclusion criteria. These studies were divided into 2 groups based on whether they pursued the increase in physical activity through technology-aided delivery of brief periods of preferred stimulation contingent on specific responses or the use of video games (exergames) and related auditory and visual stimulation. Subsequently, a narrative synthesis of the studies was provided.

**Conclusions:**

The evidence reported by the 2 groups of studies is encouraging. However, further research is needed to compare the overall applicability and impact of the intervention strategies proposed by these groups of studies.

## Introduction

### Background

People with intellectual disabilities or multiple disabilities, such as combinations of intellectual disability and motor or sensory impairments, tend to have low (minimal) levels of physical activity compared with their typical counterparts [1-6]. Some of the more frequently reported consequences of people’s reduced levels of physical activity include (1) curtailment of their interaction with the surrounding environment and of their opportunities to learn new associations and (2) weakening of their health condition in areas such as breathing, muscle tone, and blood circulation [7-11]. Lack or reduced levels of physical activity may also create a sense of dependence and helplessness, which seriously interferes with people’s acquisition of initiative and self-determination and thus with their development and social achievement [12-15].

In light of this, there is a consensus on the need to develop intervention strategies to help people with intellectual and multiple disabilities increase their level of physical activity and hence reduce or even prevent the aforementioned consequences of low physical activity levels [16,17]. Different types of intervention programs have been suggested for this purpose. A number of those programs, for example, were based on the use of staff, parents, or caregivers’ supervision and prompts for guiding the participants through various forms of activity, which could also involve the use of exercise devices (eg, treadmills and stationary bicycles) [18-23].

Other programs have relied on the use of stimulation-regulating technologies. Such technologies generally involve sensors linked to computers or virtual reality systems that monitor the participants’ activity engagement and respond to the engagement by delivering specific forms of stimulation aimed at motivating and enhancing it. In essence, these technologies are designed to facilitate participants’ engagement in a pleasant and motivating manner and, to a large extent, independent of staff direct and consistent guidance [24-28]. Programs based on these technologies, which have received increasing recognition over the years [29-32], seem to represent a relevant intervention option for several reasons [10,33-37].

First, ensuring stimulation delivery may be critical to promote activity motivation in people who, owing to their intellectual disabilities, (1) may fail to understand the importance of engaging in physical activity (the positive impact that engaging in physical activity may have on one’s physical condition, appearance, and well-being) and thus (2) may lack such motivation [27,38,39]. Second, the possibility of resorting to stimulation-regulating technologies to manage the intervention approach, that is, response monitoring and appropriate stimulation delivery, would (1) avoid extra demands on staff time and (2) create practical and affordable conditions for facilitating and supporting physical activity in people who need improvement in this area [26,40]. Third, programs based on stimulation-regulating technologies do not force the individual to engage in activity, but rather promote the individual’s self-determination and ultimate choice of engaging in activity [27,39,41]. This last point may be considered important because it emphasizes the programs’ respect for individual freedom while supporting the individual’s rights to rehabilitation opportunities and well-being. Moreover, free (self-determined) activity engagement is likely to prevent any experience of stress and anxiety, which could materialize in the case of strict staff supervision and repeated prompting [42-45].

### Perspective

An overview of studies that have assessed intervention programs based on stimulation-regulating technologies to promote physical activity in people with intellectual and multiple disabilities could provide practically relevant information with regard to (1) the characteristics of the participants involved in the programs, (2) the technology arrangements used to monitor the participants’ activity responses and deliver stimulation, (3) the measures used to determine the impact of the programs, and (4) the overall impact findings. Although a recent effort was reported to synthesize the evidence in this area [46], such an effort (1) focused exclusively on studies assessing the impact of programs relying on video games and (2) included only 7 studies directed at people with intellectual disability over the 2010-2021 period.

This paper provides a comprehensive picture of intervention programs that use stimulation-regulating technologies to promote forms of physical activity in people with intellectual and multiple disabilities by reviewing studies carried out between 2010 and 2021 (ie, a period of relevant innovations in the field of stimulation-regulating technologies [47-49]). Such a picture would be expected to help professionals working in the area gain a clear appreciation of (1) the applicability (potential and limits) of intervention programs based on stimulation-regulating technologies and (2) the importance of exploring new intervention options and pursuing new research initiatives.

## Methods

### Search Strategy

A systematic search was conducted following the PRISMA-ScR (Preferred Reporting Items for Systematic Reviews and Meta-Analyses extension for Scoping Reviews) [50] to identify studies that reported intervention strategies based on stimulation-regulating technologies to promote physical activity in persons with intellectual and multiple disabilities. A scoping review approach was used, as our aim was to portray the technology options being used in the area and their overall applications and reported outcomes [51]. The systematic search for articles was conducted using the following databases: PubMed, Web of Science, PsycINFO, ERIC, and CINAHL. The last 3 databases were searched using the EBSCO platform. The same free-text terms were used for each database and combined by means of Boolean logical operators (*and*, *or*) to reduce the number of nonpertinent results. The resulting search syntax for all databases was as follows: “mobility” OR “physical activity” OR “exercise” OR “passive” OR “sedentary” OR “obesity” AND “technology” OR “computer” OR “mobile” OR “digital” OR “smart” OR “wearable” OR “game” OR “exergame” AND “learning disability” OR “intellectual disability” OR “developmental disability” OR “multiple disability.” Databases and search terms were chosen based on consensus among the authors.

In an attempt to possibly find additional suitable material, the systematic search of the databases was supplemented with hand searches and a Google Scholar–based *cited by* search of the references of the articles identified through the systematic search and other literature sources dealing with stimulation-regulating technologies and physical activity in people with disabilities.

### Inclusion and Exclusion Criteria

Three basic inclusion criteria were used to select the studies for the review. First, the studies involved individuals with intellectual disability or multiple disabilities, that is, a combination of intellectual disability with additional disorders, such as sensory and motor impairments. Second, the studies used stimulation-regulating technology systems aimed at helping the participants engage in forms of physical activity such as arm or leg stretching, walking, jogging, dancing, and bicycle pedaling. All these forms of engagement required a certain level of physical exertion and thus could be viewed as physical activity or exercise. Third, the impact of the intervention with the technology systems on (1) the level of activity (frequency of responses) performed or (2) some parameters of physical functioning, such as resting heart rate and balance or leg strength, was documented through specific data. There were no restrictions in the inclusion criteria with regard to the age and level of intellectual disability of the participants or the settings in which the studies were conducted. Studies were excluded if they (1) did not meet one of the aforementioned criteria (eg, focused on participants with autism spectrum disorder [52-54]), (2) were aimed at correcting the participants’ inappropriate or problem behaviors during their activity engagement [55-57], or (3) indicated the performance of occupational and functional tasks as the primary goal of the intervention, relegating the issue of physical activity to a subordinate position with no specific attention to it [58].

### Data Extraction and Coding

A data charting form was developed by the first author (GEL) and iteratively reviewed by all authors until a consensus was achieved. In line with this form, the data extracted for each study included (1) the year in which the study was published and the country in which it was carried out, (2) the participants involved, (3) the technology and stimulation conditions available, (4) the design and sessions used (the protocol followed to assess the impact of intervention), (5) the responses (measures) recorded, and (6) the outcome. Finally, following a consensus-based approach among authors, codes were created to group the studies included in the review into 2 categories. The difference between categories was based on whether the studies pursued the increase in physical activity through (1) the delivery of brief periods (eg, 10 seconds) of preferred stimulation contingent on (occurring immediately after the performance of) specific responses, or (2) the use of active video games (exergames) with related auditory and visual stimulation (see the *Results* section).

### Interrater Agreement

Interrater agreement was checked between the first (GEL) and the last (LD) authors (1) on scoring the eligibility of the 92 full-text articles, which were downloaded after the initial screening of titles and abstracts and (2) on reporting the data extracted from the articles reviewed (see the *Results* section). The percentage of interrater agreement on the 92 full-text articles was 92%; that is, the authors agreed (provided the same score *included* or *excluded*) on 85 of the 92 articles. Consensus between the authors on the 7 articles with initial disagreement was then achieved after a brief discussion. The percentage of interrater agreement on reporting the data extracted from the articles reviewed (which was checked over the data extracted from 10 articles) was 100%.

## Results

### Overview

**Figure 1:**
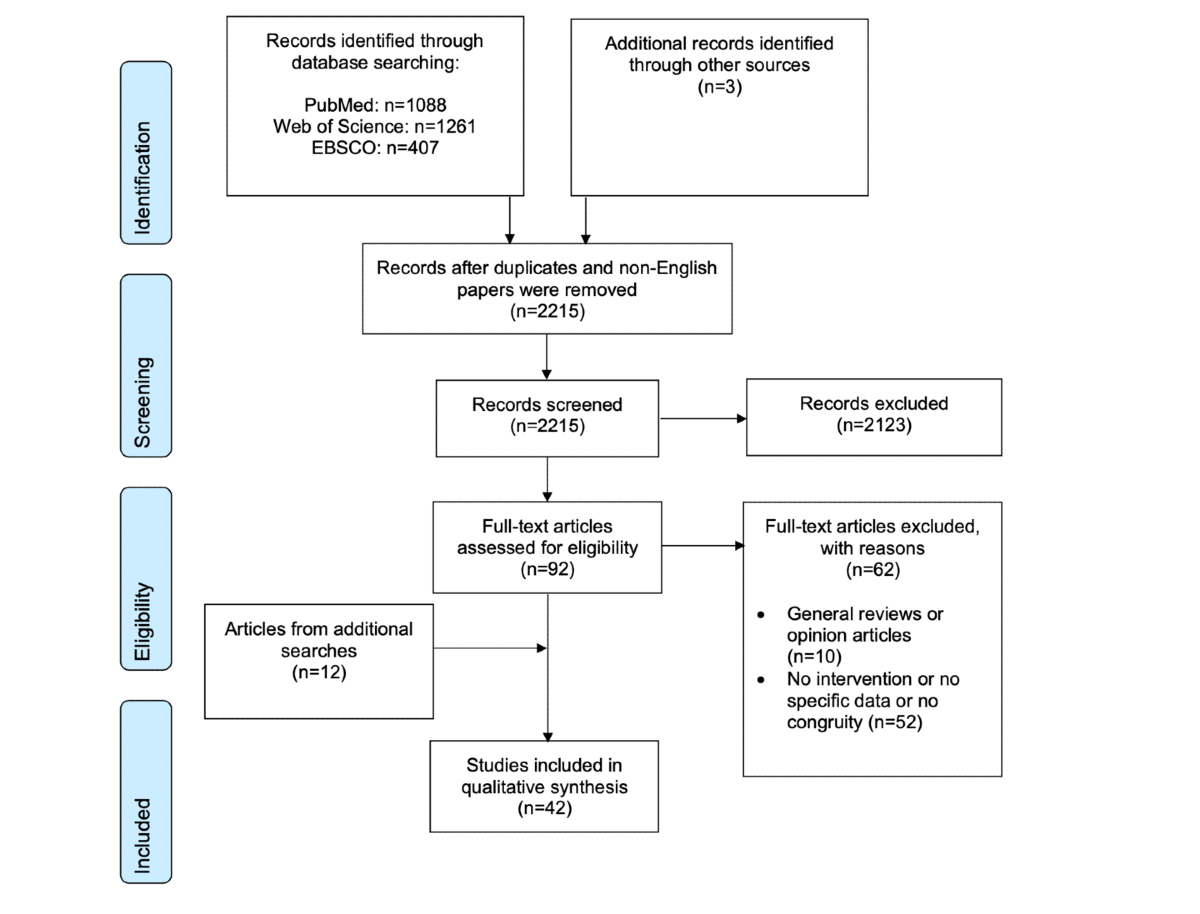
PRISMA (Preferred Reporting Items for Systematic Reviews and Meta-Analyses) flow diagram.

The database search resulted in 2756 papers. The number of papers was reduced to 2215 after duplicates and papers that were not in English were removed. Figure 1 illustrates the search process and outcomes. Initially, the titles and abstracts of the 2215 papers were screened. When the titles and abstracts were judged to be in line with the inclusion criteria, the corresponding full-text articles were downloaded. Following this process, 92 full-text articles were downloaded. The full-text articles were then read by the first (GEL) and last (LD) authors, and 30 of them were found suitable for inclusion in the review. The supplementary searches led to the finding of 12 additional articles, which were considered suitable for the review; consequently, 42 articles were finally included in the review (Figure 1).

The 42 studies (Tables 1 and 2, Multimedia Appendix 1 [10,12,27,28,32,35,37,59-78], and Multimedia Appendix 2 [26,33,34,36,47,79-88]) were conducted in Italy (n=15, 36%), Taiwan (n=14, 33%), the United States (n=5, 12%), Chile (n=1, 2%), Egypt (n=1, 2%), France (n=1, 2%), Hong Kong (n=1, 2%), Israel (n=1, 2%), New Zealand (n=1, 2%), Portugal (n=1, 2%), and the Netherlands (n=1, 2%). A total of 465 participants were included in the studies. This number concerns persons who were exposed to the intervention conditions (and excluded persons exposed to control conditions). The studies were divided into 2 groups (see the *Data Extraction and Coding* section). The first group includes studies that focused on promoting specific physical activity responses through technology-regulated delivery of preferred stimulation contingent on those responses (eg, promoting arm stretching, ambulation, or pedaling responses by delivering brief periods of preferred stimulation immediately after the performance of those responses [27,35,37]). The second group includes studies that focused on promoting physical activity through the use of video games (exergames) and related auditory and visual stimulation (eg, Wii- or other system-supported video games involving activities such as dancing or playing sports [33,79,80]).

**Table 1:** Studies based on the use of response-contingent stimulation.

Studies and countries of origin	Participants, n (age in years)	Technology	Design	Responses (measures)	Outcome
Lancioni et al [59], Italy	5 (5.6-11.4)	Optic or pressure sensors linked to a control system	Single-subject (ABAB; baseline-intervention-baseline-intervention) design	Walker-aided step responses	Positive
Shih et al [60], Taiwan	2 (17 and 19)	Wii remote control devices linked to a mini computer and television	Single-subject (ABAB) design	Arm and leg movements	Positive
Shih et al [61], Taiwan	2 (9 and 11)	A Wii balance board linked to a mini computer and television	Single-subject (ABAB) design	Change of standing posture	Positive
Shih [62], Taiwan	2 (17 and 18)	2 Wii balance boards linked to a mini computer and television	Single-subject (ABAB) design	Walking from one Wii balance board to the other	Positive
Shih et al [35], Taiwan	2 (17 and 18)	3 Wii balance boards linked to a mini computer and television	Single-subject (ABAB) design	Walking across all Wii balance boards	Positive
Tam et al [63], New Zealand	6 (38-48)	Pressure sensors linked to electronic devices	Single-subject (multiple probe) design	Arm-hand and head movements	Mainly positive
Shih et al [64], Taiwan	4 (14-17)	Technology was as in Shih et al [35]	Single-subject (ABAB) design	Walking across the Wii balance boards	Positive
Lancioni et al [12], Italy	3 (22-42)	Optic sensors linked to a computer system	Single-subject (multiple probe) design	Right and left leg-foot lifting	Positive
Lancioni et al [65], Italy	3 (10.5-34)	Technology was as in Lancioni et al [59]	Single-subject (ABAB) design	Walker-aided ambulation	Positive
Shih et al [10], Taiwan	2 (16 and 17)	A gyration air mouse linked to a mini computer and television	Single-subject (ABAB) design	Body movements	Positive
Stasolla and Caffò [66], Italy	2 (12 and 17)	Wobble and optic sensors linked to a control device	Single-subject (multiple probe) design	Object manipulation, walker-aided ambulation, indices of happiness, and stereotypies	Positive
Chang et al [67], Taiwan	2 (16 and 17)	Technology was as in Shih et al [10]	Single-subject (ABAB) design	Pedaling	Positive
Shih and Chiu [68], Taiwan	2 (16 and 17)	A dance pad linked to a mini computer and television	Single-subject (multiple probe) design	In-place walking	Positive
Lin and Chang [69], Taiwan	2 (3.9 and 4.1)	A sensor area, a webcam, and a computer	Single-subject (ABAB) design	Feet lifting	Positive
Chang et al [70], Taiwan	4 (10-18)	Technology was as in Shih et al [10]	Single-subject (ABAB) design	Walking	Positive
Lancioni et al [71], Italy	2 (19 and 38)	Optic, wobble and pressure sensors linked to a computer	Single-subject (extended ABAB) design	Arm-hand stretching and standing	Positive
Lancioni et al [72], Italy	9 (10-29)	Technology was as in Lancioni et al [71]	Single-subject (ABAB or multiple probe) design	Arm-hand and body stretching	Positive
Stasolla et al [37], Italy	2 (5 and 6)	An optic sensor linked to a control system	Single-subject (extended ABAB) design	Walker-aided ambulation and indices of happiness	Positive
Lancioni et al [27], Italy	11 (18-50)	Optic sensors linked to a computer	Single-subject (ABAB) design	Leg or hand pedaling, stepping movements, and heart rates	Positive
Lancioni et al [28], Italy	6 (16-40)	Technology was as in Lancioni et al [71]	Single-subject (ABAB or multiple probe) design	Head, arm-hand and leg-foot responses	Positive
Stasolla et al [73], Italy	5 (13-17)	Technology was as in Stasolla et al [37]	Single-subject (extended ABAB) design	Walker-aided step responses and indices of happiness	Positive
Lancioni et al [74], Italy	7 (27-52)	A smartphone and cards with code identification tags	Single-subject (multiple baseline) design	Arm and body stretching and indices of satisfaction	Positive
Lancioni et al [75], Italy	7 (9-42)	A smartphone and a small panel	Single-subject (multiple probe) design	Arm, leg, and head responses, heart rates, and indices of happiness	Positive
Stasolla et al [32], Italy	6 (5.8-9.6)	Technology was as in Stasolla et al [37]	Single-subject (extended ABAB) design	Ambulation responses, indices of positive participation, and self-injurious behavior	Positive
Lancioni et al [76], Italy	7 (30-74)	Technology was as in Lancioni et al [74]	Single-subject (multiple baseline) design	Arm and body stretching, heart rates, and indices of satisfaction	Positive
Shih et al [77], Taiwan	3 (17 or 18)	A dance pad linked to a mini computer and toy cargo train	Single-subject (multiple probe) design	Walking or running responses	Positive
Lancioni et al [78], Italy	4 (24-39)	A smartphone	Single-subject (multiple baseline) design	Independent or walker-aided ambulation	Positive

**Table 2:** Studies based on the use of video games (exergames).

Studies and countries of origin	Participants, n (age in years)	Technology	Design	Responses (measures)	Outcome
Abdel Rahman [81], Egypt	15 (10-13)	Wii Fit with balance games	Pre- and posttest plus comparison with a control group	Standing balance	Positive
Lotan et al [80], Israel	20 (37-58)	GestureTek GX single camera-based video capture VR^a^ system	Pre- and posttest plus comparison with a control group	Heart rates at rest	Positive
Wuang et al [82], Taiwan	52 (7-12)	VR using Wii gaming technology	Pre- and posttest plus comparisons with 2 control groups	Motor proficiency, visual integration, and sensory integration	Positive
Berg et al [83], United States	1 (12)	VR using Wii gaming technology	Pre- and posttest assessment	Coordination, dexterity, balance, and motor proficiency	Positive
Lin and Wuang [84], Taiwan	46 (mean 15.6)	VR using Wii gaming technology	Pre- and posttest plus comparison with a control group	Muscle strength and agility performance	Positive
Salem et al [85], United States	20 (3.3-4.8)	Wii Fit and Wii sports	Pre- and posttest plus comparison with a control group	Gait speed, balance, walking, and grip strength	Partially positive
Coyle et al [26], United States	23 (19-54)	Sony Play Station’s Dance Dance Revolution and Nintendo’s Wii sports	Cross-over design	Heart rates and self-reported preferences	DDR more effective and Wii preferred
Hsu [79], Taiwan	8 (mean 17.5)	Wii Fit balance games	Pre- and posttest plus comparisons with 2 control groups	Static balance, dynamic balance, and speed strength index	Positive
Silva et al [36], Portugal	12 (18-60)	Wii Fit balance board with strength and other games	Pre- and posttest plus comparison with a control group	Balancing, running, dancing, and others	Positive
Gómez Álvarez et al [86], Chile	9 (6-12)	Wii Fit balance board with a variety of sport related games	Pre- and posttest plus comparison with a control group	Gross motor development, balance, locomotion, and manipulation	Positive
Ryuh et al [34], United States	7 (mean 20.3)	Just Dance 3 in connection with the Xbox 360 and Kinect	Alternation of control and video games	Heart rates, perceived exertion, and enjoyment	Mainly positive
McMahon et al [87], United States	4 (14-21)	VR exercise gaming headset, stationary bicycle, and computer	Single-subject (multiple probe) design	Bicycle pedaling, heart rates, and calories burned	Positive
Lau et al [33], Hong Kong	121 (8-18)	Active video games (Sport series) and the Xbox 360 Kinect	Pre- and posttest plus comparison with a control group	Body composition, physical activity level, and motor proficiency	Inconclusive
Enkelaar et al [47], The Netherlands	9 (38-68)	2×3-m Light Curtain device with light-emitting diodes and Kinect	Single-subject (multiple baseline) design	Physical activity, happiness, and well-being	Positive
Perrot et al [88], France	6 (mean 49.3)	Wii exercise games including Wii Sports and Wii Fit Plus	Pre- and posttest plus comparison with a control group	Muscular endurance, physical fitness, and cognitive functioning	Mainly positive

^a^VR: virtual reality.

Tables 1 and 2 provide preliminary information about the studies conducted within the 2 groups. Multimedia Appendices 1 and 2 include brief summaries for all these studies. Finally, the text presents a more detailed description of some studies. More detailed descriptions are aimed at helping the reader (1) acquire a more accurate view of the intervention strategies implemented and outcomes obtained and (2) develop ideas for new research and intervention strategies that would advance the level of knowledge available in the area.

### Studies Based on the Use of Response-Contingent Stimulation

Of the 42 studies, 27 (64%; including 112 participants; Table 1 and Multimedia Appendix 1) were conducted to promote physical activity via technology-regulated delivery of preferred stimulation contingent on specific participants’ responses [10,12,27,28,32,35,37,59-78]. The reasoning at the basis of these studies was that (1) the possibility of helping people with intellectual and multiple disabilities engage in physical activity may largely depend on the context’s ability to motivate them to do so and (2) an effective way of motivating them could involve the use of preferred stimulation contingent on responses considered functional for their physical activity [10,27,35].

As shown in Table 1 and, more specifically in Multimedia Appendix 1, the studies adopted technology solutions, which included, among others, sensors (microswitches) linked to an electronic control system and stimulation devices, and dance pads or Wii balance boards linked to a mini computer and a television set. The preferred stimulation available for the single responses targeted during the studies could include auditory, visual, and vibrotactile events. The single events could last between approximately 2 and 12 seconds [10,27,71,72,74], with the possibility of producing a continuous stimulation input if responding occurred with consistency [12,37,65,68].

For example, Lancioni et al [59] worked with 5 children aged 5.6 to 10.1 years who presented with severe to profound intellectual disability and motor and sensory impairments and tended to be passive and sedentary. The study aimed to promote walker-aided ambulation (step) responses and was conducted according to an ABAB design (a single-subject design alternating A-baseline and B-intervention phases) for 4 participants, whereas it only included an AB sequence for the fifth participant. The stimulation-regulating technology consisted of pressure sensors fixed to the children’s shoes or optic sensors fixed to the walker and an electronic control system. This system, which was linked to the sensors and stimulation devices, monitored the participants’ performance of step responses throughout the A and B phases of the study and regulated the delivery of preferred (auditory and vibrotactile) stimulation contingent on those responses during the B phases. The stimulation events set for these responses typically lasted from 3 to 5 seconds. The participants’ mean frequency of step responses during the first baseline varied between approximately 7 and 26 per 5-minute session. During the first intervention phase of the study, the frequency showed more than a 3-fold increase over the baseline levels. The frequency declined during the second baseline phase and increased again during the second intervention phase.

Shih [62] investigated the possibility of increasing the physical activity of 2 participants aged 17 and 18 years with moderate or profound intellectual disability and sedentariness. One of these participants was also obese. The technology involved 2 Wii balance boards and a control system consisting of a mini computer linked to the balance boards and a television set. The participants were to walk from one balance board to another and stand on it. This study was conducted according to an ABAB design. During the A phases, the system only recorded the number of responses (walking to and standing on a balance board) the participants performed during the 3-minute sessions. During the B phases, the system also provided the participants with 6 seconds of preferred videos and music contingent on each response. During the first baseline phase, participants had a mean of approximately 3 responses per session. During the first intervention phase, their response means increased 4 to 5 times, reaching nearly 13 and 15 per session. The frequency decreased during the second baseline and increased again during the second intervention.

Chang et al [67] worked with 2 participants aged 16 and 17 years with mild to moderate or severe intellectual disability and excessive body weight. The aim of this study was to promote the participants’ effective use of a stationary bicycle. The technology system included a sensor (air gyration mouse) fixed to a pedal of the bicycle and a mini computer linked to the air mouse and a television set. The television set served to present participants’ preferred videos and music. The study was carried out according to an ABAB design and included sessions of 3 minutes. During the baseline, the technology simply recorded the participants’ pedaling time. During the B phases, the technology also activated the participants’ preferred stimulation, contingent on their pedaling behavior. An interruption of ≥1 second in pedaling led to the interruption of the stimulation. During the first A phase, the participants’ pedaling accounted for approximately 48% and 10% of the session time. During the first intervention phase, pedaling showed a nearly 2-fold or 9-fold increase, reaching approximately 90% of the session time. The percentages decreased during the second baseline and increased again above the 90% level during the second B phase.

Stasolla et al [32] carried out a study with 6 children aged 5.8 to 9.6 years who were characterized by severe to profound intellectual disability linked to the Cornelia de Lange syndrome. The aim was to promote walker-aided ambulation in the participants. The technology system included (1) an optic sensor, which served to detect the participants’ step responses throughout the study, and (2) a control system that counted the step responses and their execution time and regulated the delivery of preferred stimulation events (eg, music, lights, and voices) during the intervention phases of the study. During these phases, the control system was set to activate one or more stimulus devices for a period of 4 seconds every time the participant completed 6 step responses within a 4-second interval. In addition to a basic ABAB design, the study also included control phases in which the stimulation was available during the sessions noncontingently; that is, independent of the participants’ step responses. The sessions lasted 5 minutes. During the first baseline phase, blocks of 6 step responses occurring within 4-second intervals averaged between approximately 3 and 6 per session. During the first intervention phase, the mean frequency of the blocks increased to approximately 24 to 30 per session. The frequency declined during the second baseline phase and increased again during the second intervention phase. During the intervention phases, the participants also experienced a reduction in problem behaviors and an increase in positive (eg, alertness and happiness) behaviors. Moreover, the data improvements observed during the intervention phases were largely lost during the control phases, in which the stimulation was freely available rather than contingent on blocks of steps performed within 4-second intervals.

Lancioni et al [75] worked with 7 participants aged 9 to 42 years who presented with moderate or severe to profound intellectual disability, motor impairments confining them to a wheelchair, and blindness or minimal residual vision. The aim was to help the participants perform responses that were functional from a physiotherapeutic standpoint and relevant in terms of physical activity. Two responses, which included arm stretching to reach and push a ball and leg-foot forward moving to push a box, were selected for each participant. A multiple probe across responses was the single-subject design used to conduct the study for each participant. Accordingly, the intervention for these responses occurred at successive times. The technology involved a smartphone whose functioning was automated via MacroDroid so that it could detect (via its proximity sensor) the participants’ responses and present a variety of auditory stimuli (eg, music and familiar voices) contingent on those responses during the intervention phases of the study. Each stimulation event lasted 10 seconds, and the sessions lasted 5 minutes. The results indicated that baseline levels of zero or near zero increased for both target responses during the intervention, reaching mean frequencies that ranged between approximately 15 and 22. During the intervention sessions, the participants also showed an increase in heart rate and in indices of happiness.

### Studies Based on the Use of Video Games (Exergames)

Of the 42 studies, 15 (36%; including 353 participants; Table 2 and Multimedia Appendix 2) were conducted to promote physical activity through the use of video games (eg, games varying from dancing to sporting events and based on systems such as Nintendo Wii and virtual reality) and the auditory and visual stimulation involved in those games [26,33,34,36,47,79-88]. Video games are considered a relevant tool that can provide adaptable, inclusive, and modifiable physical activity options to people who may be unable to access sophisticated exercise equipment and may also have low exercise motivation [46,89].

As shown in Table 2 and, more specifically, in Multimedia Appendix 2, the studies carried out in this area varied in terms of the games used, the length of time those games were played, and the type of responses (measures) they relied on to determine the impact of the games. For example, Hsu [79] investigated the capacity of Wii Fit balance games to improve the balance abilities of students with mild intellectual disabilities. Three groups of 8 participants were included in the study; that is, a Wii Fit balance game training group, a physical education group, and a sedentary activity group. The Wii Fit game training group (experimental group) received two 40-minute Wii Fit balance game sessions per week over a period of 8 weeks. The same number of sessions and weekly schedules were available for the other 2 (control) groups. The mean age of the different groups ranged from 17.4 to 17.8 years. The dynamic and static balance parameters of the experimental and control participants and their speed strength index were dependent measures. Data for the Wii Fit balance game training group showed significant pre- to postintervention differences in the duration of standing on 1 leg with the eyes closed, anteroposterior movement speed, swing area per unit time, and speed strength index. The physical education group showed significant pre- to postintervention differences in the speed strength index. The sedentary activity group did not show any significant pre- to postintervention difference.

McMahon et al [87] investigated the use of an immersive virtual reality game as a means to increase the duration and intensity of pedaling on a stationary bicycle for 4 participants with moderate intellectual disability, which in one case was combined with autism spectrum disorder. The virtual reality exercise gaming platform consisted of a Virzoom exercise bicycle and an HTC VIVE virtual reality headset. In essence, the participants could use the bicycle as a means to master various games. For example, the faster the participants pedaled on their bicycle, the faster race cars, helicopters, or other objects would move for them. They could see all these objects moving through the headset they wore during the activity sessions. The study was conducted according to a multiple probe design across participants, which meant that the baseline was extended over different periods for different participants. Sessions were set to last up to 30 minutes, but the participants could stop them at any time. The participants increased their pedaling time from approximately 3 to 6 minutes per session during baseline to between approximately 17 and 29 minutes per session during the intervention. During the intervention, the participants also (1) showed large increases in heart rate and calories burning and (2) were reported to enjoy the games available.

Lau et al [33] conducted a study involving an experimental group of 121 participants and a control group of 73 participants. The participants presented with mild intellectual disability and were aged between 8 and 18 years. The technology consisted of an Xbox 360 Kinect, and the participants in the experimental group were exposed to the intervention sessions in pairs. The sessions lasted 30 minutes and were implemented twice per week for 12 weeks. A variety of games were involved in each session, and participants could choose among those available (eg, boxing, volleyball, football, baseball, and skiing). Body composition, physical activity level, and motor proficiency were used as the outcome measures. The data showed significant changes in BMI and body fat percentage within both groups of participants during the posttest. The same trend was observed for motor proficiency. However, the effect of the intervention (after adjustment for the intervention group relative to the control group) was not statistically significant for any of the outcome measures.

## Discussion

### Principal Findings

This paper provides an overall picture of studies involving the use of stimulation-regulating technologies to promote physical activity in people with intellectual disabilities and multiple disabilities. The results of the 2 groups of studies included in the review suggest that the technologies used for the intervention programs were suitable for the participants involved and generally effective in helping them increase their physical activity or improve their physical condition. In light of the reported results and technologies, several points may be discussed. These points concern (1) the strength and characteristics of the evidence available, (2) the foundation and applicability of the intervention strategies, and (3) the practicality of the intervention strategies and related technologies. Future research directions to advance the present knowledge in this area and some limitations of the paper may also be examined.

### Strengths and Characteristics of the Evidence

Three considerations can be made with regard to this point. First, the studies using preferred stimulation contingent on participants’ responses relied on single-subject designs to determine the impact of the intervention on the level of responding (physical activity). The ABAB design (a design in which A-baseline conditions are alternated with B-intervention conditions; Table 1 and Multimedia Appendix 1) was the most frequently used. Multiple probe and multiple baseline across participants designs (designs in which the participants’ baseline phase includes different numbers of sessions or spreads over different time periods) were also used. The studies using video games mostly relied on group (randomized controlled) designs. Comparisons were carried out between the pre- and postintervention data of the experimental group, as well as between the experimental group’s data and the data of 1 or 2 control groups. On the basis of the designs used, one could argue that the evidence on the impact of the intervention reported by the studies may be considered reliable.

Second, notwithstanding the overall methodological adequacy of the studies, it may be difficult to compare and contrast the results obtained by the 2 groups; that is, the group based on response-contingent stimulation and the group based on video games. In fact, the studies in the first group typically focused on assessing whether the intervention was effective in increasing the responses targeted with contingent stimulation, assuming that this increase would in turn have beneficial effects on the participants’ physical and health conditions. The studies in the second group (except for those by Enkelaar et al [47] and McMahon et al [87]) did not assess the extent to which the intervention increased the participants’ responses. Rather, they concentrated on determining whether the intervention period would bring about benefits to participants’ physical condition (eg, balance, BMI, and muscle strength).

Third, comparisons of the results of the 2 groups of studies are difficult also because of the differences in the length of the intervention sessions and the characteristics of the participants. The length of the sessions varied between 2 and 10 minutes in the first group of studies and between 10 and 60 minutes in the second group of studies (Multimedia Appendices 1 and 2). The participants in the first group of studies often presented with severe to profound intellectual disability, which could be combined with severe and extensive motor impairments. The participants in the second group of studies were generally reported or presumed to be in the mild or moderate intellectual disability range and did not present with specific motor impairments.

### Foundation and Applicability of the Intervention Strategies

The intervention strategies used by the first group of studies were designed to deliver preferred stimulation contingent on participants’ specific activity responses, and this stimulation was assumed to (1) motivate the participants to reproduce those specific responses and thus (2) increase their activity level. Within this type of framework, the efficacy of the stimulation in promoting the acquisition and maintenance of responding is linked to its contingency value and attractive (reinforcing) power [90,91]. The more attractive the stimulation, the higher the probability that the participant would be motivated to produce the response for which the stimulation is available.

Intervention strategies based on the use of video games are also assumed to work through motivation and enjoyment. In essence, the game-specific prompting and stimulating images and auditory events are expected to facilitate the participants’ initial engagement. The additional game-related stimulation events or stimulation variations connected to the participants’ engagement are considered relevant or critical to strengthen and maintain such engagement and thus bring about an increase in the participants’ physical activity. In light of this reasoning, the game-related stimulation seems to play a role similar to that attributed to the contingent stimulation used in the first group of studies. However, notwithstanding this reasoning no assessment was reported by the second group of studies of the participants’ stimulation preferences or of whether the participants perceived the stimulation variations occurring in relation to their game engagement as truly enjoyable.

With regard to the issue of applicability, the strategies based on contingent stimulation for specific responses may be viewed as largely suitable for people with severe or profound intellectual disabilities and extensive motor or sensory impairments as well as for people with mild to moderate intellectual disabilities. For example, these strategies could be applied to help participants with different levels or combinations of disabilities to perform responses such as arm stretching and walker-supported ambulation responses or use exercise devices (1) without the need for external prompting (pressure) and (2) with apparent enjoyment of their activity engagement [27,37,68,72,74,75].

The use of video games may not be suitable for participants with severe to profound intellectual disabilities and extensive motor impairment. These participants, in fact, may possess only a narrow range of responses, which is insufficient for playing most games. Moreover, the same participants may be attracted to (motivated by) only a few types of stimuli, and these stimuli may not be included in a variety of games and should be identified through careful stimulus preference screening before the beginning of the intervention. Finally, participants with severe to profound intellectual and multiple disabilities may have serious difficulties in finding strong motivation to respond in a game situation in which much of the stimulation is available noncontingently (independent of participants’ responding) [37,73].

### Practicality of the Intervention Strategies and Related Technologies

Two considerations may be in order with regard to the practicality issue. First, the use of intervention strategies aimed at providing preferred stimulation contingent on specific participants’ responses is typically based on a multistep plan that involves (1) the identification of the responses that are feasible for the participants to perform and suitable for promoting relevant forms of physical activity, (2) the identification of stimulation events that the participants prefer (apparently enjoy), (3) the selection of sensors adequate to detect the responses and trigger a control system, and (4) the programming of the control system to deliver a brief segment of preferred stimulation any time it is triggered (any time the target responses occur). Working out this plan may be relatively demanding in terms of staff time and skills as well as technical devices. Despite its possible costs, such an approach may be critically relevant, particularly when working with people with severe to profound intellectual and multiple disabilities (see the *Studies Based on the Use of Response-Contingent Stimulation* section and Multimedia Appendix 1).

Second, the use of video games to promote physical activity might be perceived as a relatively simple approach given the availability of a wide range of games. However, in reality, it may not necessarily prove easier to arrange or more practical to manage than the use of strategies based on contingent stimulation [36,47,92]. Moreover, the fact that a variety of games are commercially available does not automatically imply that they can be considered equally suitable for all participants and that they can be implemented in any context in which the participants live [33,47].

### Future Research Directions

Future research should address several relevant issues. First, studies could be conducted to clarify different aspects of interventions using video games, such as (1) the implementation conditions (ie, the level and characteristics of staff support required to get participants involved in the games), (2) the measurement of the participants’ activity level (eg, range and frequency of responses they display during the games), and (3) variability or consistency in the activity level during the intervention period. Clarifying these aspects would help determine the procedural conditions and time costs required for the application of those games, as well as the immediate and long-term functions of the games. This information could also serve to estimate the practicality and applicability of game-based interventions in daily contexts.

Second, studies comparing interventions based on the delivery of preferred stimulation contingent on specific participants’ responses with interventions based on video games might be very important to enhance our knowledge in the area. These studies may be instrumental to determine (1) the relative value of the 2 intervention approaches with different groups of people (particularly people in the moderate range of intellectual disability) and (2) the relative cost of the approaches in terms of technology and staff involvement.

Third, in addition to measuring the increases in the participants’ levels of physical activity and related health benefits, new studies may also be focused on assessing the participants’ levels of satisfaction (indices of happiness) during the intervention sessions with the 2 types of approaches. Although some data on this issue are available [37,47,73,75], additional evidence is important to determine whether and how much these approaches can help participants experience a positive emotional condition during their activity engagement.

Fourth, social validation studies would be important to determine the opinion of staff, families, and service providers about the usability and potential of the different approaches (thus adding to early data in the area [32,73]). Social validation could be carried out by (1) showing staff, families, and service providers a few segments of the intervention sessions carried out with the 2 approaches and (2) asking them for their ratings of those segments and the technology solutions used in terms of perceived efficacy, friendliness, and overall applicability across participants and contexts [32,93].

Fifth, encouraging different research groups from different countries to be involved in new research initiatives in the area could constitute a meaningful objective to increase the generality and representativeness of the findings. This objective might be particularly relevant for studies focusing on the use of stimulation contingent on specific participants’ responses, given that the research thus far available was almost exclusively concentrated in 2 countries (Italy and Taiwan).

### Limitations

This review paper has 3 limitations. First, one might argue that a literature search restricted to articles written in English may have prevented the detection and inclusion of relevant studies published in other languages. Indeed, we have no knowledge of whether or how many potentially relevant studies were published in other languages and were not included in this review. Second, the use of free-text terms (rather than specific indexed terms) for the search of different databases might have made the search process slightly less precise (less effective in identifying all relevant articles in the area). Third, one might consider the exclusion of studies involving people with autism spectrum disorder as another limitation of this review paper. In fact, the inclusion of studies involving the participation of people with autism would have provided (1) a more comprehensive picture of the use of stimulation-regulating technologies for promoting physical activity and (2) a wider amount of evidence to determine the overall applicability and impact of those technologies within services for people with special needs. Notwithstanding the aforementioned limitations, this review paper presents a picture of the technologies and their applications and effects based on a relatively large number of studies (ie, 42 studies). This may provide credibility for the picture presented here. At the same time, it may also be a prompt for (1) extending the search to non-English articles and (2) reviewing the studies that focused on people with autism spectrum disorder and comparing their results with those obtained from people with intellectual and multiple disabilities.

### Conclusions

People with intellectual and multiple disabilities need to increase their level of physical activity, and intervention programs have been developed to help them reach this goal. This paper provides a picture of 2 groups of studies that relied on the use of stimulation-regulating technologies to work toward that goal. One group of studies sought to promote physical activity via technology-regulated delivery of preferred stimulation, contingent on specific participants’ responses. Another group of studies sought to promote physical activity through the use of video games and the auditory and visual stimulation involved in those games.

Both groups of studies reported encouraging results; however, these results cannot be easily compared and contrasted. In fact, the studies of the first group were typically focused on assessing whether the intervention was effective in increasing the responses targeted with contingent stimulation, whereas the studies of the second group mainly focused on whether the intervention would bring about benefits on the participants’ physical condition.

Future research will need to address a number of issues, including (1) the identification of the procedural conditions required for the implementation of video games; (2) comparisons between the 2 strategies in terms of impact, accessibility, practicality, and participants’ satisfaction; and (3) social validations of the 2 strategies.

## Appendix

Multimedia Appendix 1 Summary of the studies based on the use of response-contingent stimulation.

Multimedia Appendix 2 Summary of the studies based on the use of video games (Exergames).

## Abbreviations

PRISMA-ScR: Preferred Reporting Items for Systematic Reviews and Meta-Analyses extension for Scoping Reviews

## References

[1] Bartlo P, Klein PJ (2011). Physical activity benefits and needs in adults with intellectual disabilities: systematic review of the literature. Am J Intellect Dev Disabil.

[2] Dixon-Ibarra A, Driver S, Vanderbom K, Humphries K (2017). Understanding physical activity in the group home setting: a qualitative inquiry. Disabil Rehabil.

[3] Eijsvogels T, George KP, Thompson PD (2016). Cardiovascular benefits and risks across the physical activity continuum. Curr Opin Cardiol.

[4] Koritsas S, Iacono T (2016). Weight, nutrition, food choice, and physical activity in adults with intellectual disability. J Intellect Disabil Res.

[5] Queralt A, Vicente-Ortiz A, Molina-García J (2016). The physical activity patterns of adolescents with intellectual disabilities: a descriptive study. Disabil Health J.

[6] Woodmansee C, Hahne A, Imms C, Shields N (2016). Comparing participation in physical recreation activities between children with disability and children with typical development: a secondary analysis of matched data. Res Dev Disabil.

[7] Jo G, Rossow-Kimball B, Lee Y (2018). Effects of 12-week combined exercise program on self-efficacy, physical activity level, and health related physical fitness of adults with intellectual disability. J Exerc Rehabil.

[8] Lin Y, Chen C, Cho M (2012). Effectiveness of leg movement in reducing leg swelling and discomfort in lower extremities. Appl Ergon.

[9] Segizbaeva MO, Pogodin MA, Lavrova IN, Balykin MV, Aleksandrova NP (2011). [Effect of head-down tilt on respiratory responses and human inspiratory muscles activity]. Fiziol Cheloveka.

[10] Shih C, Shih C, Luo C (2013). Assisting people with disabilities in actively performing physical activities by controlling the preferred environmental stimulation with a gyration air mouse. Res Dev Disabil.

[11] Taylor MJ, Taylor D, Gamboa P, Vlaev I, Darzi A (2016). Using motion-sensor games to encourage physical activity for adults with intellectual disability. Stud Health Technol Inform.

[12] Lancioni GE, Singh NN, O'Reilly MF, Sigafoos J, Alberti G, Oliva D, Campodonico F (2013). Three non-ambulatory adults with multiple disabilities exercise foot-leg movements through microswitch-aided programs. Res Dev Disabil.

[13] Lee BK, Agarwal S, Kim HJ (2012). Influences of travel constraints on the people with disabilities’ intention to travel: an application of Seligman’s helplessness theory. Tourism Manag.

[14] Wehmeyer M, Abery BH (2013). Self-determination and choice. Intellect Dev Disabil.

[15] Wehmeyer ML, Bolding N (2001). Enhanced self-determination of adults with intellectual disability as an outcome of moving to community-based work or living environments. J Intellect Disabil Res.

[16] Bouzas S, Martínez-Lemos RI, Ayán C (2019). Effects of exercise on the physical fitness level of adults with intellectual disability: a systematic review. Disabil Rehabil.

[17] Pitchford E, Dixon-Ibarra A, Hauck JL (2018). Physical activity research in intellectual disability: a scoping review using the behavioral epidemiological framework. Am J Intellect Dev Disabil.

[18] Bassette L, Titus-Dieringer S, Zoder-Martell K, Cremeans M (2020). The use of video-based instruction to promote independent performance of physical activity skills in students with developmental disabilities in a school and community setting. Psychol Schs.

[19] Kokkoni E, Mavroudi E, Zehfroosh A, Galloway JC, Vidal R, Heinz J, Tanner HG (2020). GEARing smart environments for pediatric motor rehabilitation. J Neuroeng Rehabil.

[20] Obrusnikova I, Cavalier AR, Novak HM, Blair AE (2020). The effect of systematic prompting on the acquisition of two muscle-strengthening exercises by adults with moderate disabilities. J Behav Educ.

[21] Pinter E, Johnson JW, Boden T (2021). Using video modeling to facilitate students’ independent use of a community fitness center. Educ Treat Child.

[22] Ptomey L, Willis EA, Greene JL, Danon JC, Chumley TK, Washburn RA, Donnelly JE (2017). The feasibility of group video conferencing for promotion of physical activity in adolescents with intellectual and developmental disabilities. Am J Intellect Dev Disabil.

[23] Ptomey L, Washburn R, Lee J, Greene J, Szabo-Reed A, Sherman J, Danon J, Osborne L, Little T, Donnelly J (2019). Individual and family-based approaches to increase physical activity in adolescents with intellectual and developmental disabilities: rationale and design for an 18 month randomized trial. Contemp Clin Trials.

[24] Cai SX, Kornspan AS (2012). The use of exergaming with developmentally disabled students. Strategies.

[25] Chung AM, Harvey LA, Hassett LM (2016). Do people with intellectual disability use Nintendo Wii when placed in their home as part of a physiotherapy program? An observational study. Disabil Rehabil Assist Technol.

[26] Using technology for physical activity: a pilot study on heart rate response and game preferences. The Free Library.

[27] Lancioni GE, Singh NN, O'Reilly MF, Sigafoos J, Alberti G, Perilli V, Zimbaro C, Boccasini A, Mazzola C, Russo R (2018). Promoting physical activity in people with intellectual and multiple disabilities through a basic technology-aided program. J Intellect Disabil.

[28] Lancioni GE, Singh NN, O’Reilly MF, Sigafoos J, Campodonico F, Oliva D, Alberti G, D’amico F (2016). Using microswitch-aided programs for people with multiple disabilities to promote stimulation control and mild physical exercise. J Intellect Develop Disability.

[29] Hocking DR, Farhat H, Gavrila R, Caeyenberghs K, Shields N (2019). Do active video games improve motor function in people with developmental disabilities? A meta-analysis of randomized controlled trials. Arch Phys Med Rehabil.

[30] Mentiplay BF, FitzGerald TL, Clark RA, Bower KJ, Denehy L, Spittle AJ (2019). Do video game interventions improve motor outcomes in children with developmental coordination disorder? A systematic review using the ICF framework. BMC Pediatr.

[31] Mura G, Carta MG, Sancassiani F, Machado S, Prosperini L (2018). Active exergames to improve cognitive functioning in neurological disabilities: a systematic review and meta-analysis. Eur J Phys Rehabil Med.

[32] Stasolla F, Caffò AO, Perilli V, Albano V (2019). Supporting locomotion fluency of six children with Cornelia de Lange syndrome: awareness of microswitch responding and social validation. Techgnol Disabil.

[33] Lau PW, Wang G, Wang J (2020). Effectiveness of active video game usage on body composition, physical activity level and motor proficiency in children with intellectual disability. J Appl Res Intellect Disabil.

[34] Ryuh YJ, Chen C, Pan Z, Gadke DL, Elmore-Staton L, Pan C, Cosgriff A (2022). Promoting physical activity through exergaming in young adults with intellectual disabilities: a pilot study. Int J Dev Disabil.

[35] Shih C, Chung C, Shih C, Chen L (2011). Enabling people with developmental disabilities to actively follow simple instructions and perform designated physical activities according to simple instructions with Nintendo Wii Balance Boards by controlling environmental stimulation. Res Dev Disabil.

[36] Silva V, Campos C, Sá A, Cavadas M, Pinto J, Simões P, Machado S, Murillo-Rodríguez E, Barbosa-Rocha N (2017). Wii-based exercise program to improve physical fitness, motor proficiency and functional mobility in adults with Down syndrome. J Intellect Disabil Res.

[37] Stasolla F, Caffò AO, Perilli V, Boccasini A, Stella A, Damiani R, Albano V, Damato C (2017). A microswitch-based program for promoting initial ambulation responses: an evaluation with two girls with multiple disabilities. J Appl Behav Anal.

[38] Bossink L, van der Putten AA, Vlaskamp C (2017). Understanding low levels of physical activity in people with intellectual disabilities: a systematic review to identify barriers and facilitators. Res Dev Disabil.

[39] Michalsen H, Wangberg SC, Anke A, Hartvigsen G, Jaccheri L, Arntzen C (2020). Family members and health care workers' perspectives on motivational factors of participation in physical activity for people with intellectual disability: a qualitative study. J Intellect Disabil Res.

[40] McMahon A, McMahon DD (2016). Exercise technology interventions and individuals with IDD. Division Autism Develop Disabilities Online J.

[41] Embregts PJ, van Oorsouw WM, Wintels SC, van Delden RW, Evers V, Reidsma D (2019). Comparing a playful interactive product to watching television: an exploratory study for people with profound intellectual and multiple disabilities. J Intellect Develop Disability.

[42] Hill EE, Zack E, Battaglini C, Viru M, Viru A, Hackney AC (2014). Exercise and circulating Cortisol levels: the intensity threshold effect. J Endocrinol Invest.

[43] Hill K, Gardiner PA, Cavalheri V, Jenkins SC, Healy GN (2015). Physical activity and sedentary behaviour: applying lessons to chronic obstructive pulmonary disease. Intern Med J.

[44] Hillier A, Murphy D, Ferrara C (2011). A pilot study: short-term reduction in salivary cortisol following low level physical exercise and relaxation among adolescents and young adults on the autism spectrum. Stress Health.

[45] Russell VA, Zigmond MJ, Dimatelis JJ, Daniels WM, Mabandla MV (2014). The interaction between stress and exercise, and its impact on brain function. Metab Brain Dis.

[46] Suárez-Iglesias D, Martínez-de-Quel O, Marín Moldes JR, Ayán Pérez C (2021). Effects of videogaming on the physical, mental health, and cognitive function of people with intellectual disability: a systematic review of randomized controlled trials. Games Health J.

[47] Enkelaar L, Oosterom-Calo R, Zhou D, Nijhof N, Barakova E, Sterkenburg P (2021). The LEDs move pilot study: the Light Curtain and physical activity and well-being among people with visual and intellectual disabilities. J Intellect Disabil Res.

[48] Prosperini L, Tomassini V, Castelli L, Tacchino A, Brichetto G, Cattaneo D, Solaro CM (2021). Exergames for balance dysfunction in neurological disability: a meta-analysis with meta-regression. J Neurol.

[49] Stephenson J, Limbrick L (2015). A review of the use of touch-screen mobile devices by people with developmental disabilities. J Autism Dev Disord.

[50] Tricco AC, Lillie E, Zarin W, O'Brien KK, Colquhoun H, Levac D, Moher D, Peters MD, Horsley T, Weeks L, Hempel S, Akl EA, Chang C, McGowan J, Stewart L, Hartling L, Aldcroft A, Wilson MG, Garritty C, Lewin S, Godfrey CM, Macdonald MT, Langlois EV, Soares-Weiser K, Moriarty J, Clifford T, Tunçalp Ö, Straus SE (2018). PRISMA extension for scoping reviews (PRISMA-ScR): checklist and explanation. Ann Intern Med.

[51] Munn Z, Peters MD, Stern C, Tufanaru C, McArthur A, Aromataris E (2018). Systematic review or scoping review? Guidance for authors when choosing between a systematic or scoping review approach. BMC Med Res Methodol.

[52] Dickinson K, Place M (2014). A randomised control trial of the impact of a computer-based activity programme upon the fitness of children with autism. Autism Res Treat.

[53] Rafiei Milajerdi H, Sheikh M, Najafabadi MG, Saghaei B, Naghdi N, Dewey D (2021). The effects of physical activity and exergaming on motor skills and executive functions in children with autism spectrum disorder. Games Health J.

[54] Savage MN, Taber-Doughty T, Brodhead MT, Bouck EC (2018). Increasing physical activity for adults with autism spectrum disorder: comparing in-person and technology delivered praise. Res Dev Disabil.

[55] Lancioni GE, Singh NN, O'Reilly MF, Sigafoos J, Oliva D, Campodonico F, Alberti G, Lang R (2014). Persons with multiple disabilities exercise a complex response scheme to counter incorrect head and shoulder positions via a microswitch-aided program. J Intellect Develop Disability.

[56] Lancioni GE, Singh NN, O'Reilly MF, Sigafoos J, Perilli V, Campodonico F, Marchiani P, Lang R (2015). Persons with multiple disabilities engage in stimulus choice and postural control with the support of a technology-aided program. Behav Modif.

[57] Perilli V, Stasolla F, Caffò AO, Albano V, D’Amico F (2018). Microswitch-cluster technology for promoting occupation and reducing hand biting of six adolescents with fragile X syndrome: new evidence and social rating. J Dev Phys Disabil.

[58] Lancioni GE, Singh NN, O'Reilly MF, Sigafoos J, Alberti G, Campodonico F, Perilli V, Chiariello V, Zimbaro C (2017). A technology-aided program to support basic occupational engagement and mobility in persons with multiple disabilities. Front Public Health.

[59] Lancioni GE, Singh NN, O'Reilly MF, Sigafoos J, Oliva D, Smaldone A, La Martire ML, Stasolla F, Castagnaro F, Groeneweg J (2010). Promoting ambulation responses among children with multiple disabilities through walkers and microswitches with contingent stimuli. Res Dev Disabil.

[60] Shih C, Chang M, Shih C (2010). A limb action detector enabling people with multiple disabilities to control environmental stimulation through limb action with a Nintendo Wii Remote Controller. Res Dev Disabil.

[61] Shih C, Shih C, Chiang M (2010). A new standing posture detector to enable people with multiple disabilities to control environmental stimulation by changing their standing posture through a commercial Wii Balance Board. Res Dev Disabil.

[62] Shih C (2011). A standing location detector enabling people with developmental disabilities to control environmental stimulation through simple physical activities with Nintendo Wii Balance Boards. Res Dev Disabil.

[63] Tam GM, Phillips KJ, Mudford OC (2011). Teaching individuals with profound multiple disabilities to access preferred stimuli with multiple microswitches. Res Dev Disabil.

[64] Shih C, Chen L, Shih C (2012). Assisting people with disabilities to actively improve their collaborative physical activities with Nintendo Wii Balance Boards by controlling environmental stimulation. Res Dev Disabil.

[65] Lancioni GE, Singh NN, O'Reilly MF, Sigafoos J, Oliva D, Campodonico F, Buono S (2013). Walker devices and microswitch technology to enhance assisted indoor ambulation by persons with multiple disabilities: three single-case studies. Res Dev Disabil.

[66] Stasolla F, Caffò AO (2013). Promoting adaptive behaviors by two girls with Rett syndrome through a microswitch-based program. Res Autism Spectrum Disorders.

[67] Chang M, Shih C, Lin Y (2014). Encouraging obese students with intellectual disabilities to engage in pedaling an exercise bike by using an air mouse combined with preferred environmental stimulation. Res Dev Disabil.

[68] Shih C, Chiu Y (2014). Assisting obese students with intellectual disabilities to actively perform the activity of walking in place using a dance pad to control their preferred environmental stimulation. Res Dev Disabil.

[69] Lin C, Chang Y (2015). Interactive augmented reality using Scratch 2.0 to improve physical activities for children with developmental disabilities. Res Dev Disabil.

[70] Chang C, Chang M, Shih C (2016). Encouraging overweight students with intellectual disability to actively perform walking activity using an air mouse combined with preferred stimulation. Res Dev Disabil.

[71] Lancioni GE, Singh NN, O'Reilly MF, Sigafoos J, Alberti G, Campodonico F (2016). Case studies of technology-aided interventions to promote hand reaching and standing or basic ambulation in persons with multiple disabilities. Percept Mot Skills.

[72] Lancioni GE, Singh NN, O'Reilly MF, Sigafoos J, Alberti G, Perilli V, Campodonico F (2017). Promoting functional activity engagement in people with multiple disabilities through the use of microswitch-aided programs. Front Public Health.

[73] Stasolla F, Caffò AO, Perilli V, Boccasini A, Damiani R, D’Amico F (2017). Fostering locomotion fluency of five adolescents with rett syndrome through a microswitch-based program: contingency awareness and social rating. J Dev Phys Disabil.

[74] Lancioni GE, O’Reilly MF, Sigafoos J, Alberti G, Campodonico F, Chiariello V (2019). Promoting occupational engagement and personal satisfaction in people with neurodevelopmental disorders via a smartphone-based intervention. Adv Neurodev Disord.

[75] Lancioni GE, Singh NN, O’Reilly MF, Sigafoos J, Alberti G, Campodonico F, Tedone R, Quaranta S, Caffò AO (2018). Non-ambulatory people with intellectual disabilities practice functional arm, leg or head responses via a smartphone-based program. J Dev Phys Disabil.

[76] Lancioni G, Singh NN, O’Reilly MF, Sigafoos J, Grillo G, Campodonico F, Alberti G, Schillaci D, Zagaria T (2020). A smartphone-based intervention to enhance functional occupation and mood in people with neurodevelopmental disorders: a research extension. Life Span Disability.

[77] Shih C, Lai M, Chang M, Chang C (2020). Encouraging overweight students with intellectual disability to engage in walking/running by using a dance pad combined with a LEGO® Train. Int J Disability Develop Educ.

[78] Lancioni GE, Singh NN, O’Reilly MF, Sigafoos J, Alberti G, Chiariello V, Desideri L (2021). Use of everyday technology to promote ambulation in people with intellectual and multiple disabilities. Technol Disabil.

[79] Hsu T (2016). Effects of Wii Fit(®) balance game training on the balance ability of students with intellectual disabilities. J Phys Ther Sci.

[80] Lotan M, Yalon-Chamovitz S, Weiss PL (2010). Virtual reality as means to improve physical fitness of individuals at a severe level of intellectual and developmental disability. Res Dev Disabil.

[81] Samia A, Rahman A (2010). Efficacy of virtual reality-based therapy on balance in children with Down syndrome. World Appl Sci J.

[82] Wuang Y, Chiang C, Su C, Wang C (2011). Effectiveness of virtual reality using Wii gaming technology in children with Down syndrome. Res Dev Disabil.

[83] Berg P, Becker T, Martian A, Primrose KD, Wingen J (2012). Motor control outcomes following Nintendo Wii use by a child with Down syndrome. Pediatr Phys Ther.

[84] Lin H, Wuang Y (2012). Strength and agility training in adolescents with Down syndrome: a randomized controlled trial. Res Dev Disabil.

[85] Salem Y, Gropack SJ, Coffin D, Godwin EM (2012). Effectiveness of a low-cost virtual reality system for children with developmental delay: a preliminary randomised single-blind controlled trial. Physiotherapy.

[86] Gómez Álvarez N, Venegas Mortecinos A, Zapata Rodríguez V, López Fontanilla M, Maudier Vásquez M, Pavez-Adasme G, Hemández-Mosqueira C (2018). Effect of an intervention based on virtual reality on motor development and postural control in children with Down Syndrome. Rev Chil Pediatr.

[87] McMahon DD, Barrio B, McMahon AK, Tutt K, Firestone J (2019). Virtual reality exercise games for high school students with intellectual and developmental disabilities. J Spec Educ Technol.

[88] Perrot A, Maillot P, Le Foulon A, Rebillat A-S (2021). Effect of exergaming on physical fitness, functional mobility, and cognitive functioning in adults with down syndrome. Am J Intellect Dev Disabil.

[89] Prena K, Sherry JL (2018). Parental perspectives on video game genre preferences and motivations of children with Down syndrome. J Enabling Technol.

[90] Catania AC (2013). Learning (5th edition).

[91] Kazdin A (2012). Behavior Modification in Applied Settings.

[92] Vonstad EK, Su X, Vereijken B, Bach K, Nilsen JH (2020). Comparison of a deep learning-based pose estimation system to marker-based and kinect systems in exergaming for balance training. Sensors (Basel).

[93] Worthen D, Luiselli JK (2019). Comparative effects and social validation of support strategies to promote mindfulness practices among high school students. Child Family Behav Ther.

