# Supplementary material for: Programs Using Stimulation-Regulating Technologies to Promote Physical Activity in People With Intellectual and Multiple Disabilities: Scoping Review
Source: JMIR Rehabil Assist Technol. 2022 Apr 7;9(2):e35217. doi: 10.2196/35217 (PMC9031065; doi:10.2196/35217)
Supplement: Multimedia Appendix 1 [file rehab_v9i2e35217_app1.docx]

## MULTIMEDIA APPENDIX 1

Summary of the Studies Based on the Use of Response-contingent Stimulation.

| **Studies and countries of origin** | **Participants** | **Technology and stimulation** | **Design and sessions** | **Responses (measures)** | **Outcome** |
| --- | --- | --- | --- | --- | --- |
| Lancioni et al. (2010), Italy [61] | 5 participants with severe to profound intellectual disability and motor and sensory impairments.  Age: 5.6-11.4 years | Optic or pressure sensors to detect the participants’ walker-aided step responses and a control system that provided 3-5 s of preferred stimulation at each step response | 4 participants were exposed to an ABAB design (with A representing baseline and B intervention phases), while the fifth participant received an AB sequence. There were at least 56 intervention sessions. Sessions lasted 5 min | Walker-aided step responses | The frequency of responses during the intervention (B) phases had at least a threefold increase over the baseline (A) levels |
| Shih et al. (2010), Taiwan [62] | 2 participants with mild or unspecified intellectual disability, and motor and other impairments.  Age: 17 and 19 years | Wii remote controller devices to detect arm and leg responses and a mini computer connected to a TV providing 6 s of preferred stimulation after each response | Each participant was exposed to an ABAB design. There were 102 intervention sessions. Sessions lasted 6 min | Arm and leg movement responses | The frequency of responses during the intervention phases had at least a threefold increase over the baseline levels |
| Shih et al. (2010), Taiwan [63] | 2 participants with moderate or severe intellectual disability and motor impairment.  Age: 9 and 11 years | A Wii balance board to detect the participants’ responses and a mini computer connected to a TV functioning as in Shih et al. [62] | Each participant was exposed to an ABAB design. There were 150 intervention sessions. Sessions lasted 5 min | Change of standing posture | The frequency of posture changes during the intervention phases was more than double the baseline frequency |
| Shih (2011), Taiwan [64] | 2 participants with moderate or profound intellectual disability; one was also obese.  Age: 17 and 18 years | 2 Wii balance boards to detect the participants’ walking responses with the rest of the technology and stimulation conditions as in Shih et al. [62] | Each participant was exposed to an ABAB design. There were 114 intervention sessions. Sessions lasted 3 min | Walking from one Wii balance board to the other and standing on each board | Mean response frequencies during the intervention phases were three or four times higher than those of the baseline phases |
| Shih et al. (2011), Taiwan [35] | 2 participants with moderate or profound intellectual disability.  Age: 17 and 18 years | 3 Wii balance boards to detect the participants’ walking responses with the rest of the technology and stimulation conditions as in Shih et al. [62] | Each participant was exposed to an ABAB design. There were 114 intervention sessions. Sessions lasted 3 min | Walking across all Wii balance boards and standing on them | Mean response frequencies during the intervention phases increased about or more than tenfold compared to the baseline levels |
| Tam et al. (2011), New Zealand [65] | 6 participants with profound intellectual disability and motor and sensory impairments.  Age: 38-48 years | Pressure sensors to detect participants arm-hand and head responses and activate electronic devices producing preferred stimulation contingent on the responses (and continuing as long as the responses) | Each participant was exposed to a multiple probe design across 2 responses (with at least 16 intervention sessions) and a subsequent choice phase. Sessions lasted 10 min | Arm-hand and head movement responses | 4 participants increased the duration of the 2 target responses from low values during baseline to above 150 s per session during the intervention phases. 2 participants had a large increase in only one of the responses |
| Shih et al. (2012), Taiwan [66] | 4 participants with moderate, severe or profound intellectual disability.  Age: 14-17 years | Technology and stimulation were as in Shih et al. [35]. Participants were exposed to the study in pairs and the stimulation for walking to the right Wii balance board occurred when both members of the pair produced the response | Each pair of participants was exposed to an ABAB design. There were 123 intervention sessions. Sessions lasted 3 min | Walking of each pair across the Wii balance boards and standing on them | Mean response frequencies during the first intervention phase increased about or more than nine times over the first baseline levels. The frequencies were stable during the second intervention phase |
| Lancioni et al. (2013), Italy [12] | 3 participants with profound intellectual disability and motor and sensory impairments.  Age: 22-42 years | Optic sensors that detected leg-foot lifting responses and triggered a computer system to present 8 s of preferred stimulation contingent on each response | Each participant was exposed to a multiple probe design across 2 responses. There were at least 165 intervention sessions. Sessions lasted 5 or 10 min | Right and left leg-foot lifting responses from a sitting position and indices of positive involvement (happiness) | Response frequencies during intervention were three to five times higher than the baseline values. Indices of positive involvement also increased largely |
| Lancioni et al. (2013), Italy [67] | 3 participants with severe to profound intellectual disability and motor and sensory impairments.  Age: 10.5-34 years | Technology and stimulation conditions were comparable to those used by Lancioni et al. [61] | Each participant was exposed to an ABAB design. There were at least 358 intervention sessions. Sessions lasted between about 2 and 4 min | Walker-aided ambulation responses (i.e., single steps or forward pushes with both feet) | Response frequencies during intervention were significantly higher than during baseline for all 3 participants |
| Shih et al. (2013), Taiwan [10] | 2 participants with moderate or severe intellectual disability and obesity.  Age: 16 and 17 years | A gyration air mouse in the participants’ pocket detected any body movement they made. The rest of the technology was comparable to that used by Shih et al. [62]. 2 s of preferred stimulation followed any body movement detected by the air gyration mouse | Each participant was exposed to an ABAB design. There were 117 intervention sessions. Sessions lasted 3 min | Body movements activating the air gyration mouse | Mean body movement time during the intervention phases was at least five times higher than the body movement time of the baseline phases |
| Stasolla & Caffò (2013), Italy  [68] | 2 participants with Rett syndrome and profound intellectual disability.  Age: 12 and 17 years | Wobble sensors to detect object manipulations and optic sensors to detect walker-aided step responses. The sensors were linked to a control device that provided preferred stimulation (i.e., 10 s after each manipulation response and 3 s after each sequence of 4 steps) | Each participant was exposed to a multiple probe design across responses. There were about 90 intervention sessions on object manipulation and as many on walker-assisted ambulation. Sessions lasted 5 min | Object manipulation responses, walker-aided step responses, indices of happiness, and stereotypies | Mean frequencies of object manipulation and step responses during the intervention phases were at least three times higher than the frequencies during the baseline phases. During the intervention phases, indices of happiness increased and stereotypies declined |
| Chang et al. (2014), Taiwan [69] | 2 participants with mild to moderate or severe intellectual disability and excessive body weight.  Age: 16 and 17 years | Air gyration mouse detecting pedaling responses linked to a mini computer and TV. Pedaling triggered the TV display of preferred stimulation.  A pause of 1 s or longer in the pedaling led to the interruption of the stimulation | Each participant was exposed to an ABAB design. There were 20 intervention sessions. Sessions lasted 3 min | Pedaling responses on a stationary bicycle | Pedaling occurred for more than 90% of the session time during the intervention phases and less than 15% or slightly above 50% of the session time during the baseline phases |
| Shih & Chiu (2014), Taiwan [70] | 2 participants with mild to moderate or severe intellectual disability and excessive body weight.  Age: 16 and 17 years | A dance pad to detect in-place walking steps linked to a mini computer and a TV. Walking responses triggered the presentation of preferred stimulation. A pause in walking led to the interruption of the stimulation | A multiple probe design across participants was used to introduce the intervention. There were 11 intervention sessions. Sessions lasted 5 min | In-place walking responses | Mean frequencies of walking responses during intervention were at least five times higher than those recorded during baseline |
| Lin & Chang (2015), Taiwan [71] | 2 participants with mild intellectual or multiple disabilities.  Mean age: 4 years | A sensor area, a webcam, and a computer. When the participants’ response of lifting the feet was recorded, preferred cartoons appeared on the computer screen | Each participant was exposed to an ABAB design. There were 36 intervention sessions. Sessions lasted 3 min | Feet lifting responses | Mean frequencies of responses during intervention were at least three times higher than the frequencies recorded during baseline |
| Chang et al. (2016), Taiwan [72] | 4 participants with moderate or severe intellectual disability and excessive body weight or obesity.  Age: 10-18 years | Air gyration mouse at the participants’ calf to detect their walking responses linked to a mini computer and a TV. Walking responses triggered the presentation of preferred stimulation. A pause in walking led to an interruption in the stimulation | Each participant was exposed to an ABAB design. There were 126 intervention sessions. Sessions lasted 3-5 min | Walking responses | Mean frequencies of walking responses during intervention were between about two and nine times higher than the frequencies during baseline |
| Lancioni et al. (2016), Italy [73] | 2 participants with severe to profound intellectual disability and motor and sensory impairments.  Age: 19 and 38 years | Optic, wobble and pressure sensors to detect arm-hand stretching and standing, and a computer. Hand responses and standing led the computer to present preferred stimulation for up to 12 s | Each participant was exposed to an extended ABAB design. Eventually, the stimulation following the arm-hand responses lasted the preset time only if the participants were standing for that time. There were at least 274 intervention sessions. Sessions lasted 5 min | Arm-hand stretching and standing responses | The participants largely increased their arm-hand responding and their standing time during the intervention phases (standing reached levels of about or more than 80% of the session time) |
| Lancioni et al. (2017), Italy [74] | 9 participants with severe to profound intellectual disability and motor and sensory impairments.  Age: 10-29 years | Various types of sensors (e.g., optic, wobble, and pressure devices) to detect the participants’ arm-hand and body movement responses and a computer to present preferred stimulation. A 10-s stimulation period followed each response | Participants were exposed to an ABAB design or a multiple probe across responses design. There were at least 114 intervention sessions. Sessions lasted 5 min | Arm-hand and body stretching responses | Mean response frequencies during intervention were typically more than three or four times higher than the frequencies during baseline |
| Stasolla et al. (2017), Italy [37] | 2 participants with severe to profound intellectual disability.  Age: 5 and 6 years | An optic sensor to detect the participants’ walker-aided step responses, and a control system that provided 3 s of preferred stimulation at each step response | Participants were exposed to an extended ABAB design that also included non-contingent stimulation. There were at least 157 intervention sessions. Sessions lasted 5 min | Walker-aided step responses and indices of happiness | Mean step frequencies and indices of happiness during the intervention phases were much (significantly) higher than those observed during the baseline or the non-contingent stimulation phases |
| Lancioni et al. (2018), Italy [27] | 11 participants with severe to profound intellectual disability and motor and sensory impairments.  Age: 18-50 years | Optic sensors were (a) fixed to a static bicycle, stepper, and hand-pedaling kit to detect the participants responses and (b) linked to a computer system. The computer presented 1-3 s of preferred stimulation for each (pedaling or stepping) response | Each participant was exposed to an ABAB design. There were at least 115 intervention sessions. Sessions lasted 5 min | Leg or hand pedaling responses, stepping responses, and heart rates | All participants had statistically significant response increases during the intervention phases. Heart rates also increased with 9 participants displaying rates reflecting moderate-intensity physical activity |
| Lancioni et al. (2018), Italy [28] | 6 participants with severe to profound intellectual disability and motor and sensory impairments.  Age: 16-40 years | Technology and stimulation conditions were as in Lancioni et al. [74] | Participants were exposed to an ABAB design or a multiple probe across responses design. There were at least 138 intervention sessions. Sessions lasted 5 or 10 min | Head, arm-hand and leg-foot (stretching) responses | Mean response frequencies during the intervention phases were typically more than three or four times higher than the frequencies during baseline |
| Stasolla et al. (2018), Italy [75] | 5 participants with Rett syndrome and profound intellectual disability.  Age: 13-17 years | An optic sensor to detect walker-aided step responses was linked to a control device that provided preferred stimulation (i.e., for 3 s after each sequence of 4 steps) | Participants were exposed to an extended ABAB design that also included non-contingent stimulation. There were 140 intervention sessions. Sessions lasted 5 min | Walker-aided step responses and indices of happiness | Mean response frequencies and indices of happiness during the intervention phases were much (significantly) higher than those observed during the baseline or the non-contingent stimulation phases |
| Lancioni et al. (2019), Italy [76] | 7 participants with moderate to severe or severe to profound intellectual disability and visual or visual and motor impairments.  Age: 27-52 years | A smartphone fixed in an elevated box and cards with identification tags. Every time a card was brought to the box and touched the back of the smartphone this provided 10 s of preferred stimulation | A non-concurrent multiple baseline design across participants was used to introduce the intervention. There were at least 112 intervention sessions. Sessions lasted 5 min | Arm and body stretching to bring the cards to the elevated box, heart rates, and indices of personal satisfaction | The intervention increased the frequency of arm-body stretching responses of at least two to three times. Increases in heart rate and personal satisfaction were also recorded |
| Lancioni et al. (2019), Italy [77] | 7 participants with moderate or severe to profound intellectual disability and visual and motor impairments.  Age: 9-42 years | A smartphone behind a panel or at the side of the wheelchair’s headrest to detect arm or leg stretching responses or head raising. Following each response the smartphone delivered 10 s of preferred stimulation | Each participant was exposed to a multiple probe design across 2 responses. There were at least 193 intervention sessions. Sessions lasted 5 min | Arm, leg, and head stretching or raising responses, heart rates, and indices of happiness | The intervention increased the frequency of arm, leg and head responses of about six times or more. Increases in heart rates and indices of happiness were also recorded |
| Stasolla et al. (2019), Italy [32] | 6 participants with Cornelia de Lange syndrome and severe to profound intellectual disabilities.  Age: 5.8-9.6 years | An optic sensor to detect the participants’ walker-aided step responses, and a control system attached to the walker that provided 4 s of preferred stimulation following a sequence of 6 steps | Each participant was exposed to a design that alternated baseline and intervention phases with phases in which stimulation was non-contingent. There were 100 intervention sessions. Sessions lasted 5 min | Step (ambulation) responses, indices of positive participation, and self-injurious behavior | Mean step response frequencies and indices of positive participation during the intervention phases were much (significantly) higher than those observed during the baseline or non-contingent stimulation phases. Self-injurious behavior declined largely during the intervention phases |
| Lancioni et al. (2020), Italy [78] | 7 participants with moderate or severe to profound intellectual disability and motor impairments.  Age: 30-74 years | Technology and stimulation conditions were as in Lancioni et al. [76] | A non-concurrent multiple baseline design across participants was used to introduce the intervention. There were at least 52 intervention sessions. Sessions lasted 5 min | Arm and body stretching to bring cards to an elevated box, heart rates, and indices of personal satisfaction | The intervention increased the frequency of arm-body stretching responses of at least nine times. Increases in heart rates and personal satisfaction were also recorded |
| Shih et al. (2020), Taiwan [79] | 3 participants with mild or moderate intellectual disability.  Age: 17 or 18 years | A dance pad serving to detect walking and running responses was linked to a minicomputer and LEGO cargo train (stimulation device). The train initially moved at a constant speed as the participant had valid walking and then increased its speeds as the participant changed walking into running | A multiple probe design across participants was used to introduce the intervention. There were at least 15 intervention sessions. Sessions lasted 5 min | Walking or running step responses | The frequency of walking/running steps during the intervention increased more than five times over the baseline levels when the train speed was constant. The increase was much larger when the train speed varied according to the participant’s stepping speed |
| Lancioni et al. (2021), Italy [80] | 4 participants with severe to profound intellectual disability and sensory or sensory and motor impairments.  Age: 24-39 years | Smartphone fixed to the participants’ leg to record their step responses and trigger preferred stimulation. 3-4 s stimulation was delivered after each step | A non-concurrent multiple baseline design across participants was used to introduce the intervention. There were at least 102 intervention sessions. Sessions lasted 5 min | Independent or walker-aided step responses | The intervention mean frequencies of step responses increased two or three times over the baseline levels |

APPENDIX B. Summary of the Studies Based on the Use of Video Games (Exergames)

| **Studies and countries of origin** | **Participants** | **Technology and stimulation** | **Design and sessions** | **Responses (measures)** | **Outcome** |
| --- | --- | --- | --- | --- | --- |
| Abdel Rahman (2010), Egypt [81] | 15 participants with Down syndrome and mild to moderate intellectual disability.  Age: 10-13 years | Wii-Fit with balance games involving Wii balance boards and game-related auditory and visual stimulation | Pre- and post-test plus comparison with a control group. The 15 participants received 2 sessions a week over 6 weeks. Sessions involved 3 5-min games separated by 5-min intervals | Standing balance | The 15 participants’ post-test scores showed significant improvement over their pre-test scores as well as the post-test scores of the control group |
| Lotan et al. (2010), Israel [60] | 20 participants with severe intellectual and developmental disabilities.  Age: 37-58 years | GestureTek GX single camera-based video capture VR system presenting various games, and game-related auditory and visual stimulation | Pre- and post-test plus comparison with a control group. The 20 participants received 3 sessions a week over 8 weeks. Sessions lasted 30 min | Heart rates at rest | A significant reduction in heart rates at rest at the post-test was reported for the 20 participants (experimental group) but not for the control group |
| Wuang et al. (2011), Taiwan [82] | 52 participants with Down syndrome and unspecified (presumably mild or moderate) intellectual disability.  Age: 7-12 years | Virtual reality using Wii gaming technology with Wii Sport games, and game-related auditory and visual stimulation | Pre- and post-test plus comparisons with 2 control groups. The 52 participants received 2 sessions a week over 24 weeks. Sessions lasted 60 min | Motor proficiency, visual integration, and sensory integration | Participants using the Wii Sport games had significantly greater post-test changes than the control groups on motor proficiency, visual-integrative abilities, and sensory integrative functioning |
| Berg et al. (2012), USA [83] | 1 participant with Down syndrome and unspecified (presumably mild or moderate) intellectual disability.  Age: 12 years | Virtual reality using Wii gaming technology with various Wii sport games, and game-related auditory and visual stimulation | Pre- and post-test assessment. The participant typically received 4 sessions a week for 8 weeks. Sessions lasted 20 min or longer | Coordination, dexterity, balance, and motor proficiency | The post-test showed significant improvement in upper-limb coordination, manual dexterity, balance, and postural stability |
| Lin & Wuang (2012),  Taiwan [84] | 46 participants with Down syndrome and mild to moderate intellectual disability.  Mean age: 15.6 years | Virtual reality using Wii gaming technology with Wii Sports games and game-related auditory and visual stimulation | Pre- and post-test plus comparison with a control group. The 46 participants received 3 sessions a week over 6 weeks. Sessions lasted 20 min | Muscle strength and agility performance | The 46 participants’ post-test muscle strength and agility performance improved significantly over the pre-test measures and the post-test measures of the control group |
| Salem et al. (2012), USA [85] | 20 participants with unspecified (presumably mild or moderate) intellectual disability.  Age: 39-58 months | Wii Fit and Wii Sports focusing on balance, walking and strength and including auditory and visual stimulation | Pre- and post-test plus comparison with a control group. The 20 participants received 2 sessions a week over 10 weeks. Sessions lasted 30 min | A variety of measures including gait speed, balance, walking, and grip strength | Significant improvement over the control group was observed during the post-test with regard to balance and grip strength |
| Coyle et al. (2016), USA [26] | 23 participants with unspecified (presumably mild or moderate) intellectual and developmental disabilities and excessive body weight.  Age: 19-54 years | Sony Play Station’s DDR and Nintendo’s Wii Sports, with game-related auditory and visual stimulation | Cross-over design with participants exposed to each of the game systems in different order. Sessions lasted 45 min and alternated rest and activity periods | Heart rates and subjective, self-reported evaluation of the two types of games | The DDR game playing was more effective in increasing heart rates. Participants seemed to enjoy both types of games with a preference for the Wii games |
| Hsu (2016), Taiwan [59] | 8 participants with mild intellectual disability.  Mean age: 17.5 years | Wii Fit balance games with game-specific auditory and visual stimulation | Pre- and post-test plus comparisons with 2 control groups. The 8 participants received 2 sessions a week over 8 weeks. Sessions lasted 40 min | Static balance, dynamic balance, and speed strength index | The 8 participants’ post-test scores showed significant improvement on each of the response measures. No such improvement occurred in the control groups |
| Silva et al. (2017), Portugal [36] | 12 participants with Down syndrome and unspecified (presumably mild or moderate) intellectual disability.  Age: 18-60 years | Wii Fit balance board with strength games as well as sport related and dancing games, and game-related auditory and visual stimulation | Pre- and post-test plus comparison with a control group. The 12 participants received 3 sessions a week for a total of up to 22 sessions. Sessions lasted 60 min | A variety of measures including, among others, balancing, running, and dancing | The 12 participants’ post-test scores showed significant improvement in physical fitness, functional mobility, and motor proficiency. Only partial changes occurred in the control group |
| Gómez Álvarez et al. (2018), Chile [86] | 9 participants with Down syndrome and unspecified (presumably mild or moderate) intellectual disability.  Age: 6-12 years | Wii Fit balance board with a variety of sport related games, and game-related auditory and visual stimulation | Pre- and post-test plus comparison with a control group. The 9 participants received 2 sessions a week for 5 weeks. Sessions lasted 20 min | Gross motor development, balance, locomotion, and manipulation measures | The 9 participants showed significant post-test improvement on gross motor development and manipulation. Their post-test motor development scores were significantly better than those of the control group |
| Ryuh et al. (2019), USA [34] | 7 participants with mild to moderate intellectual disability.  Mean age: 20.3 years | Just Dance 3® in connection with the Xbox 360® and Kinect accessory, and game-related auditory and visual stimulation | A condition in which a control session was followed by a video game session and a condition in which a control week was followed by a week with video games. Sessions lasted 10 min | Heart rates, perceived exertion, and physical activity enjoyment | Data showed an increase (a) in all measures during the first condition, and (b) in heart rates and physical activity enjoyment during the second condition |
| McMahon et al. (2020), USA [87] | 4 participants with moderate intellectual and developmental disabilities.  Age: 14-21 years | VR exercise gaming headset, stationary bicycle, and computer. Bicycle pedaling controlled the types of stimulation the participants received through their headset | A multiple probe design across participants was used to introduce the intervention with games. There were 6-10 intervention sessions. Sessions could last up to 30 min | Bicycle pedaling exercise duration, heart rates, and calories burned | The mean pedaling time during the intervention was about three to eight times longer than the baseline pedaling time. Intervention also led to higher heart rates and calories burning. Participants were reported to prefer the intervention over the baseline sessions |
| Lau et al. (2020), Hong Kong [33] | 121 participants with mild intellectual disability.  Age: 8-18 years | Active video games (Sport series) and the Xbox 360 Kinect system and game-related auditory and visual stimulation | Pre- and post-test plus comparison with a control group. The 121 participants received 2 sessions per week over a period of 12 weeks. Sessions lasted 30 min | Body composition, physical activity level, and motor proficiency | The 121 participants’ post-test showed improvement over the pre-test. Yet, the changes on their body composition, physical activity level, and motor proficiency were not significantly different from those observed in the control group |
| Enkelaar et al. (2021), The Netherlands [47] | 9 participants with moderate or severe intellectual disability and visual impairments.  Age: 38-68 years | 2 x 3 m Light Curtain device with light-emitting diodes and Kinect presenting a variety of video games and game-related auditory and visual stimulation | A multiple baseline design across participants was used to introduce the intervention with games. The participants received seven intervention sessions. Sessions lasted between 15 and 30 min | Physical activity, excitement (happiness), and well-being | The 9 participants’ physical activity and positive excitement were higher during the intervention sessions (i.e., when engaging with the Light Curtain) than during baseline (i.e., with care-as-usual activities) |
| Perrot et al. (2021), France [88] | 6 participants with Down syndrome and unspecified (presumably mild or moderate) intellectual disability.  Mean age: 49.3 years | Wii exercise games including Wii Sports as well as Wii Fit Plus with the use of Wii balance boards, and related auditory and visual stimulation | Pre- and post-test plus a comparison with a control group. The 6 participants received 2 sessions a week over 12 weeks. Sessions lasted 60 min | Muscular endurance, physical fitness, and cognitive functioning | The 6 participants’ post-test showed significant improvement on muscular endurance and physical fitness. No such improvement occurred in the control group |
