# Supplementary material for: Programs Using Stimulation-Regulating Technologies to Promote Physical Activity in People With Intellectual and Multiple Disabilities: Scoping Review
Source: JMIR Rehabil Assist Technol. 2022 Apr 7;9(2):e35217. doi: 10.2196/35217 (PMC9031065; doi:10.2196/35217)
Supplement: Multimedia Appendix 2 [file rehab_v9i2e35217_app2.docx]

## MULTIMEDIA APPENDIX 2

Summary of the Studies Based on the Use of Video Games (Exergames)

| **Studies and countries of origin** | **Participants** | **Technology and stimulation** | **Design and sessions** | **Responses (measures)** | **Outcome** |
| --- | --- | --- | --- | --- | --- |
| Abdel Rahman (2010), Egypt [81] | 15 participants with Down syndrome and mild to moderate intellectual disability.  Age: 10-13 years | Wii-Fit with balance games involving Wii balance boards and game-related auditory and visual stimulation | Pre- and post-test plus comparison with a control group. The 15 participants received 2 sessions a week over 6 weeks. Sessions involved 3 5-min games separated by 5-min intervals | Standing balance | The 15 participants’ post-test scores showed significant improvement over their pre-test scores as well as the post-test scores of the control group |
| Lotan et al. (2010), Israel [60] | 20 participants with severe intellectual and developmental disabilities.  Age: 37-58 years | GestureTek GX single camera-based video capture VR system presenting various games, and game-related auditory and visual stimulation | Pre- and post-test plus comparison with a control group. The 20 participants received 3 sessions a week over 8 weeks. Sessions lasted 30 min | Heart rates at rest | A significant reduction in heart rates at rest at the post-test was reported for the 20 participants (experimental group) but not for the control group |
| Wuang et al. (2011), Taiwan [82] | 52 participants with Down syndrome and unspecified (presumably mild or moderate) intellectual disability.  Age: 7-12 years | Virtual reality using Wii gaming technology with Wii Sport games, and game-related auditory and visual stimulation | Pre- and post-test plus comparisons with 2 control groups. The 52 participants received 2 sessions a week over 24 weeks. Sessions lasted 60 min | Motor proficiency, visual integration, and sensory integration | Participants using the Wii Sport games had significantly greater post-test changes than the control groups on motor proficiency, visual-integrative abilities, and sensory integrative functioning |
| Berg et al. (2012), USA [83] | 1 participant with Down syndrome and unspecified (presumably mild or moderate) intellectual disability.  Age: 12 years | Virtual reality using Wii gaming technology with various Wii sport games, and game-related auditory and visual stimulation | Pre- and post-test assessment. The participant typically received 4 sessions a week for 8 weeks. Sessions lasted 20 min or longer | Coordination, dexterity, balance, and motor proficiency | The post-test showed significant improvement in upper-limb coordination, manual dexterity, balance, and postural stability |
| Lin & Wuang (2012),  Taiwan [84] | 46 participants with Down syndrome and mild to moderate intellectual disability.  Mean age: 15.6 years | Virtual reality using Wii gaming technology with Wii Sports games and game-related auditory and visual stimulation | Pre- and post-test plus comparison with a control group. The 46 participants received 3 sessions a week over 6 weeks. Sessions lasted 20 min | Muscle strength and agility performance | The 46 participants’ post-test muscle strength and agility performance improved significantly over the pre-test measures and the post-test measures of the control group |
| Salem et al. (2012), USA [85] | 20 participants with unspecified (presumably mild or moderate) intellectual disability.  Age: 39-58 months | Wii Fit and Wii Sports focusing on balance, walking and strength and including auditory and visual stimulation | Pre- and post-test plus comparison with a control group. The 20 participants received 2 sessions a week over 10 weeks. Sessions lasted 30 min | A variety of measures including gait speed, balance, walking, and grip strength | Significant improvement over the control group was observed during the post-test with regard to balance and grip strength |
| Coyle et al. (2016), USA [26] | 23 participants with unspecified (presumably mild or moderate) intellectual and developmental disabilities and excessive body weight.  Age: 19-54 years | Sony Play Station’s DDR and Nintendo’s Wii Sports, with game-related auditory and visual stimulation | Cross-over design with participants exposed to each of the game systems in different order. Sessions lasted 45 min and alternated rest and activity periods | Heart rates and subjective, self-reported evaluation of the two types of games | The DDR game playing was more effective in increasing heart rates. Participants seemed to enjoy both types of games with a preference for the Wii games |
| Hsu (2016), Taiwan [59] | 8 participants with mild intellectual disability.  Mean age: 17.5 years | Wii Fit balance games with game-specific auditory and visual stimulation | Pre- and post-test plus comparisons with 2 control groups. The 8 participants received 2 sessions a week over 8 weeks. Sessions lasted 40 min | Static balance, dynamic balance, and speed strength index | The 8 participants’ post-test scores showed significant improvement on each of the response measures. No such improvement occurred in the control groups |
| Silva et al. (2017), Portugal [36] | 12 participants with Down syndrome and unspecified (presumably mild or moderate) intellectual disability.  Age: 18-60 years | Wii Fit balance board with strength games as well as sport related and dancing games, and game-related auditory and visual stimulation | Pre- and post-test plus comparison with a control group. The 12 participants received 3 sessions a week for a total of up to 22 sessions. Sessions lasted 60 min | A variety of measures including, among others, balancing, running, and dancing | The 12 participants’ post-test scores showed significant improvement in physical fitness, functional mobility, and motor proficiency. Only partial changes occurred in the control group |
| Gómez Álvarez et al. (2018), Chile [86] | 9 participants with Down syndrome and unspecified (presumably mild or moderate) intellectual disability.  Age: 6-12 years | Wii Fit balance board with a variety of sport related games, and game-related auditory and visual stimulation | Pre- and post-test plus comparison with a control group. The 9 participants received 2 sessions a week for 5 weeks. Sessions lasted 20 min | Gross motor development, balance, locomotion, and manipulation measures | The 9 participants showed significant post-test improvement on gross motor development and manipulation. Their post-test motor development scores were significantly better than those of the control group |
| Ryuh et al. (2019), USA [34] | 7 participants with mild to moderate intellectual disability.  Mean age: 20.3 years | Just Dance 3® in connection with the Xbox 360® and Kinect accessory, and game-related auditory and visual stimulation | A condition in which a control session was followed by a video game session and a condition in which a control week was followed by a week with video games. Sessions lasted 10 min | Heart rates, perceived exertion, and physical activity enjoyment | Data showed an increase (a) in all measures during the first condition, and (b) in heart rates and physical activity enjoyment during the second condition |
| McMahon et al. (2020), USA [87] | 4 participants with moderate intellectual and developmental disabilities.  Age: 14-21 years | VR exercise gaming headset, stationary bicycle, and computer. Bicycle pedaling controlled the types of stimulation the participants received through their headset | A multiple probe design across participants was used to introduce the intervention with games. There were 6-10 intervention sessions. Sessions could last up to 30 min | Bicycle pedaling exercise duration, heart rates, and calories burned | The mean pedaling time during the intervention was about three to eight times longer than the baseline pedaling time. Intervention also led to higher heart rates and calories burning. Participants were reported to prefer the intervention over the baseline sessions |
| Lau et al. (2020), Hong Kong [33] | 121 participants with mild intellectual disability.  Age: 8-18 years | Active video games (Sport series) and the Xbox 360 Kinect system and game-related auditory and visual stimulation | Pre- and post-test plus comparison with a control group. The 121 participants received 2 sessions per week over a period of 12 weeks. Sessions lasted 30 min | Body composition, physical activity level, and motor proficiency | The 121 participants’ post-test showed improvement over the pre-test. Yet, the changes on their body composition, physical activity level, and motor proficiency were not significantly different from those observed in the control group |
| Enkelaar et al. (2021), The Netherlands [47] | 9 participants with moderate or severe intellectual disability and visual impairments.  Age: 38-68 years | 2 x 3 m Light Curtain device with light-emitting diodes and Kinect presenting a variety of video games and game-related auditory and visual stimulation | A multiple baseline design across participants was used to introduce the intervention with games. The participants received seven intervention sessions. Sessions lasted between 15 and 30 min | Physical activity, excitement (happiness), and well-being | The 9 participants’ physical activity and positive excitement were higher during the intervention sessions (i.e., when engaging with the Light Curtain) than during baseline (i.e., with care-as-usual activities) |
| Perrot et al. (2021), France [88] | 6 participants with Down syndrome and unspecified (presumably mild or moderate) intellectual disability.  Mean age: 49.3 years | Wii exercise games including Wii Sports as well as Wii Fit Plus with the use of Wii balance boards, and related auditory and visual stimulation | Pre- and post-test plus a comparison with a control group. The 6 participants received 2 sessions a week over 12 weeks. Sessions lasted 60 min | Muscular endurance, physical fitness, and cognitive functioning | The 6 participants’ post-test showed significant improvement on muscular endurance and physical fitness. No such improvement occurred in the control group |
